# Effectiveness of Public Health Interventions in Reducing Morbidity and Mortality during Heat Episodes: a Structured Review

**DOI:** 10.3390/ijerph7030991

**Published:** 2010-03-10

**Authors:** Kate L Bassil, Donald C Cole

**Affiliations:** 1 Faculty of Health Sciences, Simon Fraser University, 8888 University Drive, Burnaby, BC V5A 1S6, Canada; 2 Dalla Lana School of Public Health, University of Toronto, 155 College Street, Toronto, ON M5T 3M7, Canada; E-Mail: donald.cole@utoronto.ca

**Keywords:** heat stress disorders, interventions, environment, morbidity, mortality, public health

## Abstract

Increasing concern over the impact of hot weather on health has fostered the development of public health interventions to reduce heat-related health impacts. However, evidence of the effectiveness of such interventions is rarely cited for justification. Our objective was to review peer-reviewed and grey literature evaluating interventions aimed at reducing morbidity and/or mortality in populations during hot weather episodes. Among studies considering public risk perceptions, most respondents were aware when an extreme heat episode was occurring but did not necessarily change their practices, primarily due to a lack of self-perception as vulnerable and confusion about the appropriate actions to be taken. Among studies of health outcomes during and following heat episodes, studies were suggestive of positive impacts in reducing morbidity and mortality. While the limited evaluative work to date suggests a positive impact of public health interventions, concern persists about whether the most vulnerable groups, like the elderly and homeless, are being adequately reached.

## 1. Introduction

The adverse effects of hot weather on health are of increasing public health concern, particularly for urban areas. With climate change, warmer climates are expected to result in higher mean summer temperatures and fluctuations will likely result in more frequent and intense heat waves and their associated health risks [1]. Environmental health practitioners and policymakers are faced with the challenge of deciding both which public health interventions are most appropriate and when and how they should be implemented at the regional and local level [2–4]. However, information regarding the effectiveness of public health interventions to reduce morbidity and mortality during heat episodes is often not brought into decision-making and requires further study [5,6].

To better equip environmental/public health practitioners and policymakers in making these important decisions, we recently undertook an examination of the types of public health interventions implemented globally during heat episodes, including media announcements, opening of cooling centres, outreach to vulnerable groups, and website bulletins [7]. The aim of this structured review is to address the question: *What is the evidence on effectiveness of public health interventions in reducing morbidity and mortality during heat episodes?*

## 2. Methods

Environmental health practitioners and policymakers were invited to provide advice throughout the project in agreement with recommendations on the process of developing systematic reviews relevant to public health policy [8,9]. Their role was both advisory, to assist us in identifying research gaps and priorities in this area, as well as resourceful, by directing us to relevant materials that may not have been detected by our search.

Two major information sources were explored: peer-reviewed literature; and grey literature, the latter including non-published sources such as conference proceedings, government documents, theses, working papers, and reports.

### 2.1. Peer-Reviewed Literature

Following initial discussions with our expert advisory group and our initial scan, we anticipated that limited information would be available in the peer-reviewed literature. As a result, our search strategy was designed to be as comprehensive as possible in an effort to capture any relevant materials. We searched the following bibliographic databases: Medline, PreMedline, and Scholars Portal. We did not include a date or other restrictions on the kinds of papers retrieved at this stage. Terms were used in the search strategy included:

At least one of: plan, planning, program, response, intervention, evaluation, response, warning, alert, watch, public health response, implementation, prevention, awareness, education, preparedness, control, measures, strategy, system, risk management, disaster management, emergency management; “And” with at least one of: heat or heat stroke or heatstroke or extreme weather or summer weather or heat wave or heat event or heat stress or heat episode or hot weather or excessive weather.

For each search the titles, abstracts, and sometimes the full text of articles were reviewed to determine relevance. Articles that were selected included those that presented public health interventions used during heat episodes and considered an evaluation of effectiveness. Stringent inclusion and exclusion criteria were not applied, given the limited information encountered but we maintained a focus on interventions designed specifically for human health rather than planning, landscape ecology, or architectural literature, more broadly addressing urban form and heat load.

Reference lists of relevant articles were scanned for other relevant material. Key authors of the papers were contacted via email for additional information and direction to relevant resources. Prominent heat stress researchers, as identified in the literature, were also contacted to identify any evaluative work not identified through our search.

### 2.2. Grey Literature

Several strategies were used to capture the grey literature related to the review topic.

- Internet search engines: Similar terms to those used for the peer-review literature were applied through major search engines including Google, GoogleScholar, and Scirus to search for other non-published literature, conference proceedings, government documents, theses, working papers, and reports.
- Web-sites: Specific global and national web-sites that might include public health/heat information were visited and contacts followed-up to determine any evaluative work completed or in-progress.
- Institutional repositories: These were searched for faculty publications that may or may not be included in peer-reviewed sources, theses, and conference proceedings.
- GPO Cat/Pac and the Canadian Research Index: Here we sought policy documents, memos and reports generated by municipalities, public health units and other levels of government.

### 2.3. Focusing the Review

The majority of evaluations we encountered were evaluations of heat-health warning systems (HHWS) and their robustness as meteorological forecasts. Very few studies evaluated the effectiveness of interventions put in place as a result of warnings generated from these systems, partly due to the challenge in defining “effectiveness” of interventions during heat episodes. Possible meanings or indicators of effectiveness might include: messages actually reaching people (awareness), reported changes in individual practice, use of services (e.g., calls to information lines, visits to cooling centres), subsequent reduced morbidity and mortality. For the purpose of this review we decided to examine effectiveness in terms of two key sets of indicators:

1. Public awareness of an extreme heat episode and any subsequent changes in practices.
2. Documented changes in morbidity and mortality attributed to the implementation of public health interventions.

For studies including these indicators, a data extraction form was developed for each type to capture relevant information. For each type, a pair of reviewers extracted data and noted major strengths and limitations. These in turn informed our synthesis of findings and our ideas regarding research directions. Given the substantial heterogeneity in study design, these findings are presented in the narrative, and summarized in Table 1, which provides an illustration of the breadth of study design and methodologies. It is interesting to note that the majority of the studies are from the US and Western Europe, with a clear lack of studies in other regions, including many developing countries that are particularly vulnerable to the impacts of hot weather.

## 3. Results

### 3.1. Public Awareness and Individual Change in Practices

Earlier indirect indicators of awareness and practices were based on routinely collected data coming from services during heat episodes. For example, in Philadelphia during the summer of 2002, their phone information “Heatline” received over 2,300 calls [10]. As the summer progressed, fewer calls were received, likely due to less media attention, less advertising of the Heatline, and a reduced need for information given the acclimatization of the population over the course of the summer. A Toronto study surveyed staff involved in operating HHWS and those working with vulnerable groups as part of public health responses [5]. Respondents felt that although much of the general public were aware of a heat alert being declared this was less true for the vulnerable, elderly and socially isolated.

A recently used method to directly assess awareness and change in practices among the public is through the use of public surveys. These surveys typically take place face-to-face in a public venue, or over the phone. A postal survey was conducted after the 2003 heat wave in Portugal to assess individual heat protective measures both during hot episodes and specifically during the 2003 heat wave [11]. Knowledge of the heat warning was nearly universal (92%). In general, there were significantly better practices by those who had obtained information. However, the elderly (75+) and less-educated were less likely to heed advice, which is of major concern given these are more vulnerable groups.

A survey was conducted in France between 2005 and 2006 to assess the awareness and practices of the public during heat alerts [12]. Recall of heat alerts from radio and television broadcasts was high (74%). This awareness was associated with a relatively high level of change in practice: 63% of respondents took protective measures in 2006 *versus* 48% in 2005. All practices polled (increased hydration, closing sun-facing windows, *etc.*) showed increased uptake from 2005 to 2006, from 6–15%. Similarly, respondents reported increased efforts to support vulnerable friends and family, with 73% of respondents reported helping someone. However, only 63% of the elderly respondents reported having been helped and only 14% reported asking for help when they felt discomfort.

A recent study distributed 201 surveys to individuals in metropolitan Phoenix, Arizona, to gauge risk perception and warning response to heat episodes [13]. The majority of individuals surveyed reported that they were aware when a heat advisory was issued. However, there was variation in this awareness across different demographic categories (women more aware than men, respondents over the age of 65 years reported the highest level of awareness). Despite the nearly universal awareness of a heat advisory, it did not necessarily translate into action—less than 50% of those over 65 changed their behaviour during a heat warning. There was an elevated perception of risk among Hispanics that translated to increased response. The conclusions from this study were that while most people receive the messages, only about half of the population actually change their behaviour in response to a heat event.

These perception studies have focused on the general public; given that everyone is a risk to the health effects of heat to varying degrees it is clearly important to understand the general public’s perception and response to such interventions. However, telephone and postal surveys and face-to-face interviews that recruit participants at public places such as shopping plazas, typically in suburban rather than urban areas, do not capture important vulnerable groups like the socially isolated and homeless. More recently, there has been an increasing number of studies that examine such vulnerable groups specifically, particularly the elderly.

A telephone survey of 908 participants over the age of 65 years was conducted in four cities (Dayton, Philadelphia, Phoenix, Toronto) to assess knowledge of heat warnings [14]. Knowledge of the heat warning system was nearly universal (90%) and likely due to pervasive media coverage (primarily television). However, knowledge of the details of the message of the mitigation plans were less well understood, and few individuals actually changed practice in response. Many respondents did not believe they were vulnerable or that the messages applied to them. There was also reported confusion around the difference between ozone precautions and heat precautions.

More recently, a study in the UK interviewed seniors populations living at home (>72 years of age) to assess heat risk perception [15]. While few recognized themselves at risk they did recognize the risks and medical concerns in others. This raises interesting questions regarding the role of self-perception and the challenge in delivering targeted strategies to groups that do not actually consider themselves to be at increased risk. A Canadian study investigated heat perception in people with chronic cardiac and pulmonary disease [16]. By capturing potential participants through medical sites/clinics the researchers were able to capture this particularly vulnerable group. Interestingly, this chronically ill population did perceive themselves to be susceptible to heat and reported implementing preventive actions in response. Given their increased vulnerability, this is an encouraging finding and highlights the importance of future studies targeted at such at-risk groups.

A survey regarding awareness of the “St. Louis Operation Weather Survival” among the elderly and health service providers was conducted in 1995 [17]. Health and social service providers noted that elderly often were not concerned about heat (e.g., “I’ve lived here all my life, never had air conditioning, so why would I have a problem now?”) or not taking advantages of resources (“cooling shelters are only for really poor people”). Interviews with elderly corroborated these perceptions. Including health service providers was a particular strength of this survey, as it provided a unique perspective often not captured in heat health perception work that has important implications for the delivery of interventions, particularly to populations receiving ongoing medical care such as those residing in residential care facilities.

A study evaluating the effectiveness of the National Heatwave Plan in the UK also explored perception by health care providers and staff of regional public health units, social care inspectors, and primary care trusts [18]. Similarly, while the staff found the Heatwave Plan to be useful in preparing for heatwaves, they expressed concern over whether information was actually reaching vulnerable populations given the challenge in contacting such a large number of people. It was suggested that the definition of “vulnerable groups” be refined to focus on those most at risk; for example women over 85 years living alone.

### 3.2. Change in Health Outcomes (Morbidity and Mortality)

The other commonly used method in the public health heat interventions evaluation literature is to compare the occurrence of adverse health outcomes in time periods with and without warning systems and response plans in place. The outcome typically examined is change in mortality, with change in morbidity infrequently assessed.

One study calculated the number of lives saved and the economic benefit of warnings in reducing heat-related mortality as a result of implementing a HHWS in Philadelphia [19]. By using multiple regression to assess the relationship between excess mortality and several explanatory variables (*i.e.*, daily weather, duration of heatwave, whether a warning was called, *etc.*) the authors conclude that issuing a warning lowered daily mortality by 2.6 lives. The operational costs of running this warning system was practically at a “noise” level compared to the economic benefits (using the EPA valuation of a statistical life at $6.12 million) of saving 117 lives in three years. This is the only attempt to assign an economic value to the potential lives saved as a result of implementing a HHWS. However, there are challenges in assigning such tangible values. They may only partially reflect the full “value” of a life lost, excluding less quantifiable, intangible components such as the intrinsic value of a person to their family/community. Similarly, appropriate economic evaluation would require full costing of all interventions, at least on a marginal basis.

More commonly, studies make comparisons between different heat wave periods; typically, one where there was not a response plan in place with a subsequent heat wave when interventions were implemented. For example, research in the Czech Republic reported a decrease in mortality during the 2003 European heatwaves as compared with earlier years [20]. Part of this decrease was attributed to greater public awareness of heat warnings that were issued in the 2000s when hot weather was forecasted. This is further supported by lessons from France where fewer heat-related mortalities were reported in 2006 following the implementation of a HHWS and its affiliated interventions in 2004 [21,22]. Another study used heat-related morbidity and mortality during 1995 and 1999 heat waves in Milwaukee, Wisc. to compare heat-related mortality rates and ambulance services [23]. The lower rates in 1999 were attributed to improvements in public health response.

Similarly, another study compared the mortality occurring in heat waves during 1995 and 1999 in the Midwestern US (with a focus on Chicago and St. Louis) [24]. The authors conclude that in 1999, Chicago more successfully mitigated adverse effects than it did in 1995 (119 deaths during the 1999 heatwave compared with more than 500 deaths during the 1995 heatwave). This was attributed to improvements in public health response (in addition to characteristics of the heat wave). A key factor was also felt to be the upgrading and better performance of the electrical supply which was maintained during the 1999 heat wave, whereas it failed during the 1995 episode.

Finally, a study in St. Louis, Missouri compared mortality in the 1980 and 1995 heat waves and found higher mortality in 1980, primarily because the 1980 heat wave was more severe and longer in duration than the 1995 event [25]. A simulated model of 1980 weather conditions and 1995 population suggested the St. Louis population was more vulnerable in 1995 than in 1980 despite an increase in air conditioning availability and improved public health response. The author attributes this to increases in the “frail elderly” population over 74, and rising poverty rates among the general population as well as persons over 65 years. This highlights the challenges in attributing differences in health outcomes to public health interventions. Due to differences in heat events and often the relatively short period between events, it is unclear to what extent the mortality or morbidity reduction could be attributed to intervention efforts *versus* meteorological factors or reduced susceptibility of the population.

## 4. Synthesis

From the collected evaluation literature several common themes arise. The first is that awareness of heat events/alerts is nearly universal in the general public. However, most public surveys reported in this paper did not include the most vulnerable groups, including the homeless or “shut-in” elderly (e.g., those who stay at home with limited social interaction) so it is not clear whether these groups are aware of heat events. The uncertainty of whether public health messages actually reach the most vulnerable was one of the most commonly cited concerns in our discussions with public health practitioners. Despite some initial work focusing particularly on the elderly, such information remains incomplete in both the peer-reviewed and grey literature. Nevertheless, the importance of targeting messages and outreach strategies is apparent in the recent assessment of awareness and practices among those with cardio-pulmonary disease [16].

Related to this is the definition of “vulnerable groups” and consideration of other groups that are not commonly included within this term but are also at a higher risk for the adverse effects of heat due to greater exposure. Included would be tourists, organizers/participants of outdoor events, and individuals who work outdoors. Novel methods to both assess awareness and practices, as well as targeting these groups, need to be developed (e.g., contacting employers in parks and recreational jobs to educate and protect outdoor employees during heat events).

Another theme in our review is that although many people are aware of heat events, fewer actually change their practices in response. Some groups like the elderly and those with pre-existing illnesses may not accurately perceive the health impacts given impaired physiologic responses. It is also partly due to the general perception by many that heat is not a killer, or that the heat message only applies to small sub-populations which the interviewees do not consider themselves to be part of; this has changed somewhat since the highly publicized 1995 and 2003 heat waves in Chicago and Europe. One reason for this suggested in conversations with public health practitioners is that the media often tend to focus on stories about occupational dangers in heat (*i.e*., parks employees, roofers, *etc.*) or one of the most commonly reported, of children or pets left in cars during heat events. This focus by the media on these specific sub-populations can lead the general population to believe that the messages are not applicable to them. This has important implications for framing the content of the messages and collaboration with media partners in ensuring accurate messages are being presented to the public. It also highlights the importance of providing both broad mass media messages as well as those targeted and tailored specifically to vulnerable groups.

A final theme arising from this work is the confusion in understanding and interpreting these public health messages. One commonly cited reason for this is the overlap between summer smog and heat warnings. Many public health units issue these warnings from separate divisions, but the findings from this report suggest that there should be coordination in these messages to better and more clearly inform the public about how they should respond (e.g., turn on air conditioners to reduce heat load *versus* turning them off to reduce electrical load and air pollution from coal fired stations). Another example is that during smog days vulnerable groups are often advised to not go outside. This conflicts with messages to go to a cooling centre.

### 4.1. Challenges in Evaluating Public Health Interventions for Heat

There are several methodological challenges in assessing the effectiveness of interventions for heat. Heat episodes are rare events that have differential impacts on each affected population. This is due to a variety of factors (*i.e*., differential distributions of individual vulnerability, level of acclimatization, *etc.*), making it difficult to compare different populations in different cities in response to a heat event. Even if there is the opportunity to study the same population over different time periods, the heat events themselves vary over time due to meteorological variation. The fact that no two heat episodes are the same makes attribution of changes in health outcomes to public health interventions versus different weather conditions or different underlying populations particularly difficult.

There are usually several public health interventions included in a response plan that are implemented simultaneously. This makes it difficult to attribute any beneficial effect to one intervention over another. Furthermore, many of the interventions are aimed at encouraging changes in individual practice.

Most HHWS and their associated response plans have been implemented only recently, with a marked increase in interest following the 2003 heat waves in Europe. This is an added challenge given the relatively short-time frame available for evaluation. Studies typically use historical information, or future projections for modelling the impact of HHWS and make comparisons with health outcomes. Nevertheless, opportunities exist for working with databases such as EM-DAT (http://www.emdat.be/) and documentation of heat response plan implementation to further such research.

Finally, evaluation of environmental health interventions remains under-developed, in contrast to rigorous evaluations of vaccines, for example. The limited published research thus reflects not only some of the challenges, but also a relative under-investment in program and policy evaluation, certainly compared to the magnitude of the problem and the importance of finding effective responses to the health burden associated with climate variability.

## 5. Conclusions

While the limited evaluative work to date suggests a positive impact of public health interventions, concern persists about whether the most vulnerable groups, like the elderly and homeless, are being adequately reached. Further studies with a focus on these vulnerable groups are needed to better understand the most effective interventions and approaches that will mitigate the adverse health outcomes they experience. Investigating heat-health risk perception is one way to develop more targeted and effective communication strategies for these groups.

There are real methodological challenges in studying the effectiveness of interventions for heat, however novel methods are being used, and more recently, a combination of methods including modelling techniques and also the incorporation of qualitative data from surveys. There is an important need to continue to improve upon these research methods and increase research activity in this area.

Finally, developing a framework for evaluating public health interventions for heat is the next important step to build on the findings of the current work. These draft criteria, indicators and analytical methods could then be applied to a selection of sites that have heat interventions in place to assess their utility. Ideally resources could be allocated and evaluations could be coordinated through a national or international organization to improve our understanding of the effectiveness of interventions that are currently being used during hot weather.

## Figures and Tables

**Table 1.** Overview of Studies Included in this Review.

**Study**	**Design**	**Location**	**Population/Health outcome**
Abrahamson (2008)	Cross-sectional survey	UK	Elderly
Angus (2006)	Cross-sectional survey	Canada	Intervention/response staff
Ebi *et al.* (2004)	Economic analysis	US	Mortality
Fouillet *et al.* (2008)	Regression analysis	France	Mortality
INPES (2006)	Cross-sectional survey	France	General public
Kalkstein *et al.* (2007)	Cross-sectional survey	US	General public
Kosatsky *et al.* (2009)	Cross-sectional survey	Canada	Cardiac/pulmonary patients
Kysely & Kriz (2008)	Regression analysis	Czech Republic	Mortality
Nogueria et al (2005)	Cross-sectional survey	Portugal	General public
Palecki *et al.* (2001)	Regression analysis	US	Mortality
Sheridan (2007)	Cross-sectional survey	US & Canada	General public
Smoyer (1997)	Cross-sectional survey	US	Elderly/health care providers
Smoyer (1998)	Regression analysis	US	Mortality
Weisskopf *et al.* (2002)	Regression analysis	US	Mortality/morbidity

## References

[1] Patz JA Campbell-Lendrum D Holloway T Foley JA Impact of regional climate change on human health Nature 2005 438 310 317 16292302 10.1038/nature04188

[2] Frumkin H Hess J Luber G Malilay J McGeehin M Climate change: the public health response Am. J. Public Health 2008 98 435 445 18235058 10.2105/AJPH.2007.119362 PMC2253589

[3] Matthies F Menne B Prevention and management of health hazards related to heat waves Int. J. Circumpolar Health 2009 68 8 22 19331238 10.3402/ijch.v68i1.18293

[4] O’Neill MS Carter R Kish JK Gronlund CJ White-Newsome JL Manarolla X Zanobetti A Schwartz JD Preventing heat-related morbidity and mortality: new approaches in a changing climate Maturitas 2009 64 98 103 19748195 10.1016/j.maturitas.2009.08.005 PMC2793324

[5] Angus J An Evaluation of Toronto’s Heat Watch Warning System M.A. Thesis, University of Toronto Toronto, ON, Canada 2006

[6] Kovats RS Hajat S Heat stress and public health: a critical review Annu. Rev. Public Health 2008 29 41 55 18031221 10.1146/annurev.publhealth.29.020907.090843

[7] Bassil K Cole DC Tomic K Callaghan M What Is the Evidence on Applicability and Effectiveness of Public Health Interventions in Reducing Morbidity and Mortality during Heat Episodes? A Review for the National Collaborating Centre for Environmental Health NCCEH Vancouver, BC, Canada 2007

[8] Ebi KL Kovats RS Menne B An approach for assessing human health vulnerability and public health interventions to adapt to climate change Environ. Health Perspect 2006 114 1930 1934 17185287 10.1289/ehp.8430 PMC1764166

[9] Greenhalgh T Robert G Macfarlane F Bate P Kyriakidou O Peacock R Storylines of research in diffusion of innovation: a meta-narrative approach to systematic review Soc. Sci. Med 2005 61 417 430 15893056 10.1016/j.socscimed.2004.12.001

[10] Kalkstein LS Description of our heat/health watch-warning systems: their nature and extent and required resources Available online: http://www.health.state.mn.us/divs/eh/emergency/natural/heat/uofdelaware.html (accessed on 3 April, 2007).

[11] Nogueria PJ Paixao EJ Falcao JM Comportamentos das familias portuguesas em epocas de calor e durante a onda de calor de Agosto de 2003 [in Portuguese] Vigilancia epidemiologica 2005 23 2 18

[12] INPES (Institut National de Prévention et d’Education pour la Santé) Bilan de la vague de chaleur 2006 et actions nouvelles pour lutter contre une canicule Available online: http://www.sante.gouv.fr/htm/actu/canicule_231006/dp_bilan_vague_chaleur_2006.pdf (accessed on 21 November, 2009).

[13] Kalkstein AJ Sheridan SC The social impacts of the heat-health watch/warning system in Phoenix, Arizona: assessing the perceived risk and response of the public Int. J. Biometeorol 2007 52 43 55 17262221 10.1007/s00484-006-0073-4

[14] Sheridan SC A survey of public perception and response to heat warnings across four North American cities: an evaluation of municipal effectiveness Int. J. Biometeorol 2007 52 3 15 17024398 10.1007/s00484-006-0052-9

[15] Abrahamson V Wolf J Lorenzoni I Fenn B Kovats S Wilkinson P Adger WN Raine R Perceptions of heatwave risks to health: interview-based study of older people in London and Norwich, UK J. Public Health 2009 31 119 126 10.1093/pubmed/fdn102 19052099

[16] Kosatsky T Dufresne J Richard L Renouf A Giannetti N Bourbeau J Julien M Braidy J Sauve C Heat awareness and response among Montreal residents with chronic cardiac and pulmonary disease Can. J. Public Health 2009 100 237 240 19507730 10.1007/BF03405548 PMC6973932

[17] Smoyer KE Environmental Risk Factors in Heat Wave Mortality in St Louis PhD Dissertation, University of Minnesota Minneapolis, MN, USA 1997

[18] Johnson S Bickler G Evaluation of the Department of Health National Heatwave Plan 2007 Available online: http://www.hpa.org.uk/web/HPAwebFile/HPAweb_C/1204100449763 (Accessed on 9 February, 2010).

[19] Ebi KL Teisberg TJ Kalkstein LS Robinson L Weiher RF Heat watch/warning systems save lives—estimated costs and benefits for Philadelphia 1995–98 Am. Meteorol. Soc 2004 85 1067 1073

[20] Kysely J Kriz B Decreased impacts of the 2003 heat waves on mortality in the Czech Republic: an improved response? Int. J. Biometeorol 2008 52 733 745 18612662 10.1007/s00484-008-0166-3

[21] Fouillet A Rey G Wagner V Laaidi K Empereur-Bissonnet P Le Tertre A Frayssinet P Bessemoulin P Laurent F De Crouy-Chanel P Jougla E Hemon D Has the impact of heat waves on mortality changed in France since the European heat wave of summer 2003? A study of the 2006 heat wave Int. J. Epidemiol 2008 37 309 317 18194962 10.1093/ije/dym253 PMC2652641

[22] Toulemon L Barbieri M The mortality impact of the August 2003 heat wave in France: investigating the ‘harvesting’ effect and other long-term consequences Popul. Stud. (Camb.) 2008 62 39 53 18278672 10.1080/00324720701804249

[23] Weisskopf MG Anderson HA Foldy S Hanrahan LP Blair K Torok TJ Rumm PD Heat wave morbidity and mortality, Milwaukee, Wis, 1999 *vs* 1995: an improved response? Am. J. Public Health 2002 92 830 833 11988455 10.2105/ajph.92.5.830 PMC1447169

[24] Palecki MA Changnon SA Kunkel KE The nature and impacts of the July 1999 heat wave in the Midwestern United States: learning from the lessons of 1995 Bull. Meteorol. Soc 2001 82 1353 1367

[25] Smoyer KE A comparative analysis of heat waves and associated mortality in St. Louis, Missouri—1980 and 1995 Int. J. Biometeorol 1998 42 44 50 9780845 10.1007/s004840050082

